# Brain Volume Loss Can Occur at the Rate of Normal Aging in Patients with Multiple Sclerosis Who Are Free from Disease Activity

**DOI:** 10.3390/jcm11030523

**Published:** 2022-01-20

**Authors:** Joke Temmerman, Floris Van Der Veken, Sebastiaan Engelborghs, Kaat Guldolf, Guy Nagels, Dirk Smeets, Gert-Jan Allemeersch, Lars Costers, Marie B. D’hooghe, Anne-Marie Vanbinst, Jeroen Van Schependom, Maria Bjerke, Miguel D’haeseleer

**Affiliations:** 1 Department of Neurology, Universitair Ziekenhuis Brussel (UZ Brussel), Laarbeeklaan 101, 1090 Brussels, Belgium; joke.temmerman@uantwerpen.be (J.T.); floris.van.der.veken@vub.be (F.V.D.V.); sebastiaan.engelborghs@uzbrussel.be (S.E.); kaat.guldolf@gmail.com (K.G.); guy.nagels@uzbrussel.be (G.N.); marie.dhooghe@mscenter.be (M.B.D.); 2 Center for Neurosciences (C4N), NEUR and AIMS, Vrije Universiteit Brussel (VUB), Laarbeeklaan 103, 1090 Brussel, Belgium; dirk.smeets@icometrix.com (D.S.); lars.costers@icometrix.com (L.C.); jeroen.van.schependom@vub.be (J.V.S.); maria.bjerke@uzbrussel.be (M.B.); 3 Department of Biomedical Sciences, Institute Born-Bunge, Universiteit Antwerpen, Universiteitsplein 1, 2610 Antwerp, Belgium; 4 Department of Neurology, Onze-Lieve-Vrouw Ziekenhuis, Moorselbaan 164, 9300 Aalst, Belgium; 5 Icometrix, Kolonel Begaultlaan 1b, 3012 Leuven, Belgium; 6 Department of Radiology, Universitair Ziekenhuis Brussel (UZ Brussel), Laarbeeklaan 101, 1090 Brussels, Belgium; gert-jan.allemeersch@uzbrussel.be (G.-J.A.); annemarie.vanbinst@uzbrussel.be (A.-M.V.); 7 Nationaal Multiple Sclerose Centrum (NMSC), Vanheylenstraat 16, 1820 Melsbroek, Belgium; 8 Department of Electronics and Informatics (ETRO), Vrije Universiteit Brussel (VUB), Pleinlaan 2, 1050 Brussels, Belgium; 9 Laboratory of Clinical Neurochemistry, Department of Clinical Biology, Universitair Ziekenhuis Brussel (UZ Brussel), Laarbeeklaan 101, 1090 Brussels, Belgium

**Keywords:** multiple sclerosis, brain volume loss, NEDA-3

## Abstract

Multiple sclerosis (MS) is a chronic inflammatory demyelinating and degenerative disorder of the central nervous system. Accelerated brain volume loss (BVL) has emerged as a promising magnetic resonance imaging marker (MRI) of neurodegeneration, correlating with present and future clinical disability. We have systematically selected MS patients fulfilling ‘no evidence of disease activity-3′ (NEDA-3) criteria under high-efficacy disease-modifying treatment (DMT) from the database of two Belgian MS centers. BVL between both MRI scans demarcating the NEDA-3 period was assessed and compared with a group of prospectively recruited healthy volunteers who were matched for age and gender. Annualized whole brain volume percentage change was similar between 29 MS patients achieving NEDA-3 and 24 healthy controls (−0.25 ± 0.49 versus −0.24 ± 0.20, *p* = 0.9992; median follow-up 21 versus 33 months; respectively). In contrast, we found a mean BVL increase of 72%, as compared with the former, in a second control group of MS patients (*n* = 21) whom had been excluded from the NEDA-3 group due to disease activity (*p* = 0.1371). Our results suggest that neurodegeneration in MS can slow down to the rate of normal aging once inflammatory disease activity has been extinguished and advocate for an early introduction of high-efficacy DMT to reduce the risk of future clinical disability.

## 1. Introduction

Multiple sclerosis (MS) is a chronic demyelinating and degenerative disorder of the central nervous system (CNS) affecting nearly three million people worldwide [1]. The clinical course is usually characterized by subacute exacerbations of neurological dysfunction (termed relapses)—in most cases followed by at least partial recovery—and/or a more slowly evolving progression of disability. Autoimmune inflammatory responses play a crucial role in the formation of demyelinating lesions, which are visible as focal hyperintensities on T2-weighted magnetic resonance imaging (MRI) and constitute the pathological basis for the relapses [2]. Brain volume loss (BVL), as a surrogate of brain atrophy, has recently emerged as a popular MRI marker of neurodegeneration in MS, strongly associated with concurrent and future disability at both the physical and cognitive level [3,4]. The rate of BVL is approximately 0.05–0.3% per year in the general population, depending on the age category [5], while this process appears to advance three to five times more rapidly in subjects with MS, irrespective of the clinical phenotype [6]. Notably, shrinkage of cortical and deep gray matter structures appears to be more pronounced and relevant to clinical outcome measures, as compared with white matter volume decrease [3,4].

Current disease-modifying treatment (DMT) for MS primarily acts by reducing inflammatory processes. The term ‘no evidence of disease activity-3’ (NEDA-3) has been introduced to identify patients receiving such DMT who are completely free from new relapses, MRI lesion accrual and clinical disability worsening [7]. NEDA-3 is a composite measure with a possible twin significance: i.e., no detectable evidence of inflammation (as manifested by the absence of new relapses and MRI lesions) nor neurodegeneration (as deduced from the clinical stability). Clinical assessment, however, might be insufficient to reveal early degenerative changes, e.g., due to the initial compensatory effect of adaptive brain plasticity and/or functional reorganization [8,9,10], which stresses the need for more sensitive indicators. Indeed, the proportion of patients under DMT maintaining a NEDA-3 status considerably drops with longer follow-up duration, as compared with findings from shorter phase III trials, and the ability of the NEDA-3 label to predict future disability and sustained treatment response remains unsure [11]. To date, the question whether the rate of BVL in MS patients with NEDA-3 (and thus being totally free from visible inflammatory activity) differs from that of normal aging has not been intensively studied yet.

## 2. Materials and Methods

### 2.1. Objective

We have conducted an observational case-control study at the Universitair Ziekenhuis (UZ) Brussel and Nationaal Multiple Sclerose Centrum (NMSC) Melsbroek (Belgium). Approval was granted by the ethics committees of both centers (internal reference: 2018/417—Belgian Unique Number: 143201837786). The main goal was to compare the rate of annualized BVL between healthy controls and patients with MS who obtained a NEDA-3 status under high-efficacy DMT during the evaluation period. This objective can be translated into addressing the more relevant pathobiological question of whether neurodegeneration, under the form of accelerated BVL, continues in MS despite seemingly optimal control of inflammatory activity.

### 2.2. MS Patients

**Figure 1:**
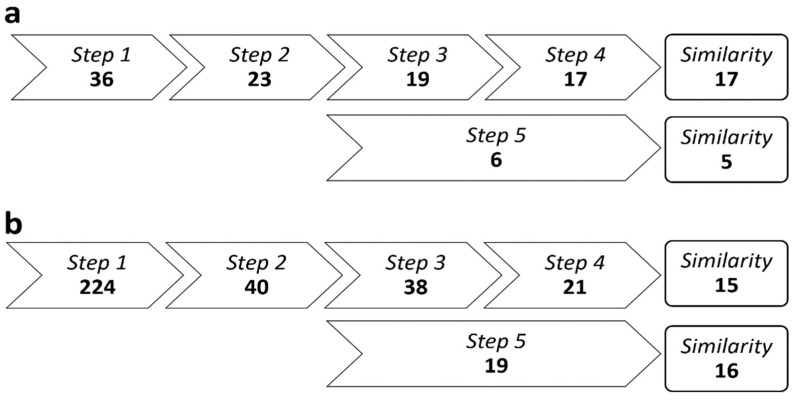
Stepwise retention procedure of multiple sclerosis patients with a ‘no evidence of disease activity-3’ (steps 1–4) versus ‘evidence of disease activity’ (step 5) status. Retention criteria are fully explained in the Methods section (in brief; step 1: matching disease-modifying treatment—step 2: suitable magnetic resonance imaging—step 3: no radiological lesion load accumulation—step 4: absence of relapses and disability worsening—step 5: identification of patients with evidence of disease activity, in parallel with steps 3 and 4, based on the number of retained subjects after step 2). Resulting number of patients after each step are displayed separately for the Universitair Ziekenhuis Brussel (**a**) and Nationaal Multiple Sclerose Centrum Melsbroek (**b**). A similarity scoring was performed by icometrix after step 5.

To form the case group, a systematic and rigorous selection procedure was applied on the complete neurological databases of the UZ Brussel and NMSC Melsbroek, in a stepwise order described hereunder and displayed in Figure 1.

Step 1 A shortlist was generated on 24 August 2018 containing all unique patients with MS who were under treatment with natalizumab, fingolimod, or ocrelizumab in one of both centers at that time. Patients who had received treatment with alemtuzumab or cladribine in the 5 preceding years (and in whom no other subsequent DMT had been administered thereafter) were also added.
Step 2 The most recent (before 24 August 2018) brain MRI including 3D T1-weighted and fluid-attenuated inversion recovery (FLAIR) images was tracked in all candidates obtained from step 1 and labeled as MRI-1. We subsequently searched for the brain MRI including 3D T1-weighted and FLAIR images which was performed the closest to the day 24 months prior to MRI-1; the latter was labeled as MRI-0. A minimum interval of 12 months between MRI-0 and MRI-1 was deemed mandatory to enable sufficient between-group BVL contrastation. Only patients who had been continuously treated with natalizumab, fingolimod, or ocrelizumab between MRI-0 and MRI-1, or those who had received alemtuzumab or cladribine prior to MRI-0 (and in whom no other DMT was administered between MRI-0 and MRI-1), were retained. The substantial disparity in retention between the two study sites (i.e., 64% versus 18%) after this step might be explained by fundamental differences with regard to the general working environment and/or facilities at both centers. The UZ Brussel is a classic academic hospital where the vast majority of patients followed at the MS unit are referred to the in-house radiology department for MRI evaluation, using a standardized protocol with 3D FLAIR and T1-weighted imaging in each subject. In contrast, the NMSC Melsbroek is a not a general hospital but a tertiary center specifically focusing on the neurological management, multidisciplinary care, and rehabilitation of individuals with MS. The latter has a larger database (which is also reflected by the higher number of patients resulting from step 1) but relies on referral to other hospitals for MRI monitoring, introducing radiological heterogeneity and thereby reducing the likelihood of meeting the inclusion criteria of step 2.
Step 3 MRI-0 was compared with MRI-1, as well as with each available intermediate brain MR. Patients were removed if, at any point throughout the follow-up period, there was (a) at least one new or increased hyperintense lesion on FLAIR imaging or (b) contrast enhancement on T1-weighted imaging.
Step 4 We identified the neurological report that chronologically preceded MRI-0 first, as well as the report that was the first to follow MRI-1 (respectively labeled as R-0 and R-1). All interim reports were screened for the occurrence of MS relapses; defined as subacute new or worsening neurological deficits, disabling and present for at least 24 h, and in the absence of fever/infection or a more appropriate alternative explanation. Expanded Disability Status Scale (EDSS) scores were recorded from R-0 and R-1 [12]. If not available in these reports, EDSS scores were reconstructed by the study team to the best of their ability based on the information in the patient records. Patients with evidence for one or more MS relapses, as well as those with significant EDSS progression, between R-0 and R-1, were not retained. The following criteria were used to define significant EDSS progression: at least 1.5 points if the R-0 score was 0, at least 1.0 points if the R-0 score was 1.0–5.0, or at least 0.5 points if the R-0 score was 5.5 or higher [13].
Step 5 In parallel, steps 1 to 4 also resulted in the identification of MS patients with evidence of disease activity (EDA) while being on high-efficacy DMT between MRI-0 and MRI-1, who served as an additional control group.

### 2.3. Healthy Controls

A group of healthy volunteers who had already undergone a 3D T1-weighted and FLAIR MRI of the brain between 2015 and 2018 (i.e., MRI-0), in the context of a separate study conducted at the UZ Brussel (*n* = 46) [14], was invited for a repeat exam (i.e., MRI-1) for the purpose of our project (original ethical approval of the REF14 study was amended). MRI-0 and MRI-1 were executed under the same methodological circumstances.

### 2.4. Brain Volume Analysis

Automated brain volume quantification was performed on the 3D T1-weighted and FLAIR images of MRI-0 and MRI-1 in each MS patient (NEDA-3 and EDA group) retrieved by our five-step selection procedure, as well in every control subject in whom a repeat brain MRI was performed, using icobrain ms (icometrix, Leuven, Belgium) software for which the method has been described earlier [15,16]. For MRI pairs stemming from real-world clinical practice, and thus potentially obtained from different scanners (i.e., the total cohort of MS patients), a similarity index was calculated as the normalized mutual information after a linear registration of the two scans. The normalized mutual information was computed as the sum of the entropy present in each image, divided by the joint entropy after linear registration. The MRI pairs were retained for final analysis only if this similarity index was larger than 0.15. This threshold is based on an experiment in which the test-retest error of icobrain ms was assessed on 155 within- and 306 between-scanner pairs acquired on the same day. The average test-retest error for intra-scanner pairs, inter-scanner pairs with good similarity, and inter-scanner pairs with poor similarity was 0.2%, 0.5%, and 1.5%, respectively (unpublished data). The difference in annualized whole brain volume change (expressed as a percentage) between MS patients with a NEDA-3 status and healthy controls was a priori selected as the primary endpoint of this study.

### 2.5. Statistics

Statistical analyses were performed with GraphPad Prism version 9.0.0 (GraphPad Software; San Diego, CA, USA). Data for which the Shapiro–Wilk test demonstrated a normal distribution were expressed as the mean (SD); median (range) values were used if this was not the case unless specified otherwise. Group differences were assessed by means of unpaired Student *t*-tests or Mann–Whitney *U* tests, where appropriate. Pearson’s *r* and Spearman’s *ρ* were calculated to measure the strength and direction of the linear relationship between variables, where appropriate. All *p* values are two-tailed, unless specified otherwise, and were declared statistically significant at the 0.05 level.

## 3. Results

### 3.1. Study Groups

Figure 1 illustrates the identification process of the MS patients with a NEDA-3 status, as described in detail above. Our search yielded 38 potential candidates, of whom 32 showed sufficient similarity between both MRI scans for BVL measurement. Eighteen of them were under DMT with natalizumab, 12 with fingolimod, and 2 with alemtuzumab. In parallel, we identified 25 MS patients with EDA, of whom 21 were retained for BVL measurement after similarity scoring. In the EDA group, 12 subjects were under DMT with natalizumab, 7 with fingolimod, and 2 with alemtuzumab. The control group consisted of 27 healthy volunteers. The demographics of the three groups are shown in Table 1.

### 3.2. Brain Volume Change

Because there was a difference of more than 10% in gender distribution and mean age between the MS patients with NEDA-3 and healthy controls (the latter reaching a trend towards statistical significance), a supplementary matching procedure was performed by a study team member (GN) who was blinded to the volumetric outcomes, using MATLAB version R2020b (MathWorks, Natick, MA, USA). Both the NEDA-3 and healthy control groups were repeatedly resampled, drawing a subset from both groups without replacement and during which three subjects were removed from each group. For each repetition of this resampling procedure, we calculated the differences in age and gender distribution between the two subsets, and then we chose those two subsets that had the smallest difference in both parameters. DMT representation in the MS patients with NEDA-3 changed to 17/11/1 for natalizumab, fingolimod, and alemtuzumab, respectively. Adjusted demographics and brain volume results are displayed in Table 2. No significant differences were found for annualized whole brain and total gray matter volume change. The interval between both MRI scans was significantly shorter in the MS patients with NEDA-3, as compared with the healthy volunteers.

BVL was less pronounced in the MS patients with NEDA-3, as compared to those with EDA (original unadjusted groups were used for statistical analyses, demographical data are displayed in Table 1), as demonstrated by the 72% difference in mean (± SD) annualized whole brain volume percentage change (−0.25 ± 0.47 versus −0.43 ± 0.63, respectively), although this result did not reach statistical significance (one-tailed *p* = 0.1371). Similar observations were made for mean (± SD) annualized total gray matter volume change (25% difference: −0.36 ± 0.63 versus −0.45 ± 0.78, one-tailed *p* = 0.3263).

### 3.3. Correlations

Significant positive correlations were noted between annualized whole brain and total gray matter volume percentage change in the total MS cohort (*n* = 53; *r* = 0.61, *p* < 0.0001), as well as in the healthy controls (*n* = 27; *r* = 0.44, *p* = 0.0462). The time interval between the two MRI exams did not correlate with annualized whole brain nor total gray matter volume percentage change in the adjusted NEDA-3 (*ρ* = 0.31, *p* = 0.1106; *ρ* = 0.28, *p* = 0.1567; respectively) and healthy control groups (*ρ* = −0.23, *p* = 0.2755; *ρ* = −0.24, *p* = 0.2660; respectively) that were matched for gender and age.

## 4. Discussion

NEDA-3 has recently been launched as an ambitious treatment goal in the clinical management of MS, striving for complete suppression of inflammatory disease activity. In patients who have obtained such a label, a significantly lower rate of BVL was demonstrated across the evaluation period, compared with their counterparts who did not [17,18], although the difference with normal aging has not been extensively investigated yet. Our study now shows a similar degree of whole brain and total gray matter volume decline in patients with MS, as measured between two MRI time points demarcating a period of NEDA-3, and a group of healthy volunteers who were matched for age and sex, suggesting that neurodegeneration can slow down to physiological levels once inflammatory disease activity has been silenced. These findings are in line with those from a prospective Norwegian cohort study—to our knowledge, the only other direct comparative trial in this field—in which 57 patients with early relapsing-remitting MS were stratified into a NEDA-3 (49%) versus EDA (51%) category based on 1 year of follow-up. The authors did not find a statistically significant increase in annualized subcortical gray matter volume loss, using the freely available FreeSurfer (Charleston, MA, USA) analytical software, in the MS patients with NEDA-3, as compared with an age- and gender-matched control group (*n* = 61) without neurological or psychiatric disease (controls had a longer radiological interval; 42 months on average), whereas the opposite was true for the MS patients with EDA [19]. Tissue-specific BVL, particularly involving the thalamus [20], has developed as a valuable marker and prognosticator of clinical MS severity but we provide the advantage of presenting whole brain volume data since, overall, global brain volume changes still seem to be more strongly associated with clinical outcome measures than regional estimates [4]. In addition, icobrain ms is one of the three quantification packages cleared by both the European Medicines Agency and the United States Food and Drug Administration and currently the sole application that has been independently validated for purposes related to MS [21]. Our conclusions are seemingly contradicted by reports revealing considerable rates of MS patients achieving NEDA-3 to have a whole BVL above the anticipated pathological threshold of 0.4% per year [22,23,24]. However, such general cut-offs are unlikely to be applicable to all participants and a substantial portion was under a lower-efficacy platform DMT regimen (i.e., interferon β or glatiramere acetate), also illustrating the heterogeneity of studies addressing this issue.

Knowledge about the behavior of other promising candidate biomarkers of neurodegeneration in MS is still limited but seems to align with the results from our study. First, elevated and correlating levels of light chain neurofilament (NF-L), a specific component of the neuronal cytoskeleton, have been found in the CSF and blood of individuals with MS [25], predicting both clinical deterioration and BVL over long-term evaluation periods [26,27,28]. Release of neurofilaments has not yet been compared between large cohorts of affected patients fulfilling NEDA-3 criteria and control subjects representing normal aging. Nonetheless, consistently low NF-L levels were observed in the serum of six Korean patients with MS who achieved a NEDA-3 status over approximately 2 years of follow-up after alemtuzumab treatment, with the majority of samples remaining below the assumed limit of normality (as generated by adding two standard deviations to the mean value of age- and gender-matched control subjects) [29]. Extension studies of the alemtuzumab clinical development program have demonstrated that the majority of patients (approximately 50–60%) reached a NEDA-3 status each year while median yearly BVL remained low during the entire follow-up period (years 1–5 in CARE-MS I: −0.59%, −0.25%, −0.19%, −0.15%, and −0.20%; years 1–5 in CARE-MS II: −0.48%, −0.22%, −0.10%, −0.19%, −0.07%; respectively) [30,31]. Second, damage to the optic nerve, which may lead to retrograde axonal decay, is virtually ubiquitous in patients with MS and may lead to extensive tissue pathology in the retina, characterized by the loss of ganglion cells and their axons, respectively organized as the ganglion cells and inner plexiform layer (GCIPL) and retinal nerve fiber layer (RNFL) [32]. Studies using optical coherence tomography, which is a non-invasive technique allowing detailed in vivo imaging of the different retinal layers, have provided consistent evidence of reduced GCIPL and RNFL thickness in patients with MS [33], irrespective of having a previous clinical history of optic neuritis or not [34,35]; and correlating with poor vision, global disability, compromised quality of life and, interestingly, also with BVL on MRI [36,37,38,39,40]. Pisa and colleagues noted a relatively preserved RNFL in 20 subjects with MS who were categorized as NEDA-3 after a 2-year evaluation period, compared with those not meeting the respective criteria (*n* = 43) [41]. In more recent work by the same team, individuals with relapsing-remitting MS obtaining a NEDA-3 status were free from significant in-group RNFL reduction over a mean evaluation period of 2.4 years (13 patients; −0.27 µm/y; 95% CI: −0.89 to 0.35 µm/y; *p* = 0.39), but strikingly, this was not the case in subjects with a pre-existing diagnosis of progressive MS yet still achieving NEDA-3 criteria (11 patients; −0.56 µm/y; 95% CI: −0.98 to −0.13 µm/y; *p* = 0.01) [42]. Both studies, however, suffered from the lack of a control group representing physiological RNFL thinning.

Our findings might help the conceptual understanding of MS pathology and prevent functional decline by advocating for a more rapid introduction of a high-efficacy DMT regimen. MS is a complex disorder with causative mechanisms that are currently incompletely understood. Relapses are attributed to peripherally activated immune cells entering the CNS and mediating a local autoimmune response against the myelin sheath [43], but the precise self-antigen is unknown and it remains a matter of debate whether and how these inflammatory processes ultimately trigger neurodegeneration. Moreover, a minority school provides reasonable arguments for a contrasting “inside-out” theory in which early non-immunological CNS dysfunction is proposed as the initiating event in slowly progressive tissue disruption, leading to inflammation and autoimmunity as secondary bystander phenomena [44]. Mechanisms of neurodegeneration remain hypothetical as well, with current insights mainly involving (a) axonal transection during inflammatory outbursts, (b) energetic/mitochondrial failure in axons after long-standing demyelination, (c) cortical damage due to meningeal aggregates of lymphoid follicles, (d) detrimental innate immune activity within the normal-appearing white matter and at the borders of chronic smoldering lesions, and (e) mechanisms related to (accelerated) biological aging [45]. It needs to be determined how a NEDA-3 status relates to these points individually, but our observation of BVL, as a marker of neurodegeneration, occurring at a comparable rate between patients with MS who are apparently free from inflammatory activity and healthy age- and gender-matched control subjects, speaks in favor of autoimmune inflammation as primum movens of this disorder. Previous randomized controlled trials and real-world observations have clearly established a relationship between DMT exposure and risk reduction for clinical deterioration in patients with a relapsing phenotype [46,47,48], particularly when the former is introduced more early in the disease course [49], and with mounting evidence supporting the choice for agents with a high- over those with a medium-efficacy profile as a first treatment option [50,51,52]. Our results add to that rationale because, at least at the group level, a short-term decrease in brain volume appears to be predictive of a worse clinical status at later follow-up times in patients with a clinically isolated syndrome and relapsing-remitting MS [53,54], while it has also been shown that the preventive effect of DMT on BVL, which appears to be more pronounced for high-efficacy agents [55], is associated with its effect on disability progression [56].

The most important limitations of the study are caused by the retrospective data collection in patients with MS and by the fact that brain volume quantification in these individuals relied on MRI scans acquired outside the standardized research setting. Prospective recruitment of MS patients with NEDA-3 status risks was difficult due to the unpredictability of the disease course and treatment response. We have tried to reduce selection bias by applying a stringent selection procedure on complete hospital databases to generate our case cohort. Variability between different scanners must be acknowledged as a potential confounder [4], but we have tried to minimize this by only retaining MRI pairs that had a similarity score above an a priori defined target, as a pragmatic compromise between retaining sufficient study power and reducing the impact of technical heterogeneity. Reliability of our data in the patients with MS is supported by the presence, as biologically expected, of a positive correlation between whole brain and gray matter volumes and by the sizeable difference in BVL between subjects classified as NEDA-3 versus those as EDA. The latter did not reach statistical significance but the volumetric decline in the MS patients with EDA may have been underestimated due to the potential presence of inflammatory edema at the time of MRI-1 (‘reverse pseudo-atrophy effect’) [3,4]. The inter-scan interval was longer in the healthy controls, as compared to the MS patients with NEDA-3, but significant correlations with brain volumes could not be found in the adjusted (gender- and age-matched) groups that were used for BVL comparisons. Sample sizes were relatively low, although, based on previously reported differences in annual percentage changes in whole brain volume [57], a minimum of 20 individuals in each group should be sufficient to address our primary endpoint, assuming 80% power and a 0.05 significance level. Notably, some disease-modifying agents licensed for MS, such as teriflunomide and fingolimod, have been associated with additional neuroprotective properties, independently from their anti-inflammatory effect [58,59], which, unfortunately, could not be assessed in this study because either the drug did not correspond to the inclusion criteria or patient numbers were considered too low to conduct a meaningful sub analysis. The occurrence of missing data (12% of the EDSS scores that were required for disability progression evaluation during step 4 of the NEDA-3 selection procedure were not directly available in the clinical reports and had to be reconstructed, see Materials and Methods) and the absence of more extensive (para)clinical information, such as serum NF-L and/or Symbol Digit Modalities Test measurements (the latter representing information processing speed) should also be taken into account as potential limitations of our work.

In summary, we present the first study in which whole BVL in MS patients with NEDA-3 is compared to normal aging using validated analytical software. Similar rates were observed in both situations suggesting that neurodegeneration in MS can slow down to physiological levels once inflammatory disease activity has been extinguished. These findings are pathophysiologically relevant, supporting the role of autoimmune inflammation as the leading event in MS, and advocate for an early introduction of high-efficacy DMT, although the latter does not guarantee a NEDA-3 status. Our methodological approach using similarity scores may also offer a novel strategy to validate the concept of accelerated BVL in patients with MS, originally based on studies performed in a controlled research environment, in the technically more challenging world of clinical routine.

## Figures and Tables

**Table 1:** Demographics of the final groups.

	NEDA-3	HC	EDA
Number of subjects	32	27	21
Age (years) ^1^	43 (10) *^,^^	48 (13) *	39 (10) ^^^
Gender (female/male–female/total)	23/9–0.72	16/11–0.59	14/7–0.67
EDSS score ^1^	3.6 (2.0) ^°^	NA	4.0 (1.0) ^°^
Disease duration (years) ^2^	12 (27) ^§^	NA	10 (35) ^§^
Current DMT duration (years) ^2^	3 (9) ^#^	NA	3 (9) ^#^
Interval between MRI exams (months) ^2^	22 (18) ^~^	32 (28)	20 (30) ^~^

^1^ Data expressed as the mean (SD). ^2^ Data expressed as the median (range). * *p* = 0.1013, ^^^ *p* = 0.2018, ^°^ *p* = 0.8156, ^§^ *p* = 0.9676, ^#^ *p* = 0.4632, *^~^ p* = 0.7550. NEDA-3: multiple sclerosis patients with no evidence of disease activity, HC: healthy controls, EDA: multiple sclerosis patients with evidence of disease activity, EDSS: Expanded Disability Status Scale, DMT: disease-modifying treatment, MRI: magnetic resonance imaging, NA: not applicable.

**Table 2:** Demographics and brain volume results of the matched groups.

	NEDA-3	HC
Number of subjects	29	24
Age (years) ^1,^*	44 (10) ^~^	46 (12) ^~^
Gender (female/male–female/total)	20/9–0.69	16/8–0.67
EDSS score ^1^	3.5 (1.9)	NA
Disease duration (years) ^2,^^	12 (6)	NA
Current DMT duration (years) ^2^	2 (9)	NA
Interval between MRI exams (months) ^2^	21 (18) ^°^	33 (28) ^°^
Annualized WBV change (%) ^1^	−0.25 (0.49) ^§^	−0.24 (0.20) ^§^
Annualized TGMV change (%) ^1^	−0.29 (0.60) ^#^	−0.29 (0.23) ^#^

^1^ Data expressed as the mean (SD). ^2^ Data expressed as the median (range). * Data were not normally distributed yet expressed as mean (SD) for reasons of uniformity with Table 1. ^^^ Data were normally distributed yet expressed as median (range) for reasons of uniformity with Table 1. ^~^ *p* = 0.4860, ^°^ *p* < 0.0001, ^§^ *p* = 0.9992, ^#^ *p* = 0.9668. NEDA-3: multiple sclerosis patients with no evidence of disease activity, HC: healthy controls, EDSS: Expanded Disability Status Scale, DMT: disease-modifying treatment, MRI: magnetic resonance imaging, WBV: whole brain volume, TGMV: total gray matter volume.

## Data Availability

The data presented in this study are available on request from the corresponding author.

## References

[1] Walton C., King R., Rechtman L., Kaye W., Leray E., Marrie R.A., Robertson N., La Rocca N., Uitdehaag B., Van Der Mei I. (2020). Rising prevalence of multiple sclerosis worldwide: Insights from the Atlas of MS, third edition. Mult. Scler. J..

[2] Dobson R., Giovannoni G. (2019). Multiple sclerosis—A review. Eur. J. Neurol..

[3] Sastre-Garriga J., Pareto D., Rovira A. (2017). Brain Atrophy in Multiple Sclerosis: Clinical Relevance and Technical Aspects. Neuroimaging Clin. N. Am..

[4] Sastre-Garriga J., Pareto D., Battaglini M., Rocca M.A., Ciccarelli O., Enzinger C., Wuerfel J., Sormani M.P., Barkhof F., on behalf of the MAGNIMS Study Group (2020). MAGNIMS consensus recommendations on the use of brain and spinal cord atrophy measures in clinical practice. Nat. Rev. Neurol..

[5] Battaglini M., Gentile G., Luchetti L., Giorgio A., Vrenken H., Barkhof F., Cover K.S., Bakshi R., Chu R., Sormani M.P. (2019). Lifespan normative data on rates of brain volume changes. Neurobiol. Aging.

[6] De Stefano N., Giorgio A., Battaglini M., Rovaris M., Sormani M.P., Barkhof F., Korteweg T., Enzinger C., Fazekas F., Calabrese M. (2010). Assessing brain atrophy rates in a large population of untreated multiple sclerosis subtypes. Neurology.

[7] Rotstein D.L., Healy B.C., Malik M.T., Chitnis T., Weiner H.L. (2015). Evaluation of No Evidence of Disease Activity in a 7-Year Longitudinal Multiple Sclerosis Cohort. JAMA Neurol..

[8] Pantano P., Mainero C., Caramia F. (2006). Functional Brain Reorganization in Multiple Sclerosis: Evidence from fMRI Studies. J. Neuroimaging.

[9] Pelletier J., Audoin B., Reuter F., Ranjeva J.-P. (2009). Plasticity in MS: From Functional Imaging to Rehabilitation. Int. MS J..

[10] Colorado R.A., Shukla K., Zhou Y., Wolinsky J.S., Narayana P.A. (2012). Multi-task functional MRI in multiple sclerosis patients without clinical disability. NeuroImage.

[11] Mayssam E.N., Eid C., Khoury S.J., Hannoun S. (2020). “No evidence of disease activity”: Is it an aspirational therapeutic goal in multiple sclerosis?. Mult. Scler. Relat. Disord..

[12] Kurtzke J.F. (1983). Rating neurologic impairment in multiple sclerosis: An expanded disability status scale (EDSS). Neurology.

[13] Rio J., Nos C., Tintore M., Tellez N., Galan I., Pelayo R., Comabella M., Montalban X. (2006). Defining the response to interferon-beta in relapsing-remitting multiple sclerosis patients. Ann. Neurol..

[14] Van Schependom J., Vidaurre D., Costers L., Sjogard M., D’Hooghe M.B., D’Haeseleer M., Wens V., De Tiège X., Goldman S., Woolrich M. (2019). Altered transient brain dynamics in multiple sclerosis: Treatment or pathology?. Hum. Brain Mapp..

[15] Jain S., Ribbens A., Sima D.M., Cambron M., De Keyser J., Wang C., Barnett M.H., Van Huffel S., Maes F., Smeets D. (2016). Two Time Point MS Lesion Segmentation in Brain MRI: An Expectation-Maximization Framework. Front. Neurosci..

[16] Smeets D., Ribbens A., Sima D.M., Cambron M., Horáková D., Jain S., Maertens A., Van Vlierberghe E., Terzopoulos V., Vanbinst A.-M. (2016). Reliable measurements of brain atrophy in individual patients with multiple sclerosis. Brain Behav..

[17] Rojas J., Sanchez F., Caro F., Miguez J., Patrucco L., Funes J., Cristiano E. (2019). Brain volume loss and no evidence of disease activity over 3 years in multiple sclerosis patients under interferon beta 1a subcutaneous treatment. J. Clin. Neurosci..

[18] Håkansson I., Tisell A., Cassel P., Blennow K., Zetterberg H., Lundberg P., Dahle C., Vrethem M., Ernerudh J. (2018). Neurofilament levels, disease activity and brain volume during follow-up in multiple sclerosis. J. Neuroinflammation.

[19] Nygaard G.O., Celius E.G., Benavent S.A.D.R., Sowa P., Gustavsen M.W., Fjell A.M., Landrø N.I., Walhovd K.B., Harbo H.F. (2015). A Longitudinal Study of Disability, Cognition and Gray Matter Atrophy in Early Multiple Sclerosis Patients According to Evidence of Disease Activity. PLoS ONE.

[20] Ontaneda D., Raza P.C., Mahajan K.R., Arnold D.L., Dwyer M.G., Gauthier S.A., Greve D.N., Harrison D.M., Henry R.G., Li D.K.B. (2021). Deep grey matter injury in multiple sclerosis: A NAIMS consensus statement. Brain.

[21] Steenwijk M.D., Amiri H., Schoonheim M.M., de Sitter A., Barkhof F., Pouwels P.J., Vrenken H. (2017). Agreement of MSmetrix with established methods for measuring cross-sectional and longitudinal brain atrophy. NeuroImage Clin..

[22] Guevara C., Garrido C., Martinez M., Farías G., Orellana P., Soruco W., Alarcón P., Diaz V., Silva C., Kempton M.J. (2019). Prospective Assessment of No Evidence of Disease Activity-4 Status in Early Disease Stages of Multiple Sclerosis in Routine Clinical Practice. Front. Neurol..

[23] Yokote H., Kamata T., Toru S., Sanjo N., Yokota T. (2018). Brain volume loss is present in Japanese multiple sclerosis patients with no evidence of disease activity. Neurol. Sci..

[24] Kappos L., de Stefano N., Freedman M.S., Cree B., Radue E.-W., Sprenger T., Sormani M.P., Smith T., Häring D.A., Meier D.P. (2016). Inclusion of brain volume loss in a revised measure of ‘no evidence of disease activity’ (NEDA-4) in relapsing–remitting multiple sclerosis. Mult. Scler. J..

[25] Khalil M., Teunissen C.E., Otto M., Piehl F., Sormani M.P., Gattringer T., Barro C., Kappos L., Comabella M., Fazekas F. (2018). Neurofilaments as biomarkers in neurological disorders. Nat. Rev. Neurol..

[26] Thebault S., Abdoli M., Fereshtehnejad S.-M., Tessier D., Tabard-Cossa V., Freedman M.S. (2020). Serum neurofilament light chain predicts long term clinical outcomes in multiple sclerosis. Sci. Rep..

[27] Barro C., Benkert P., Disanto G., Tsagkas C., Amann M., Naegelin Y., Leppert D., Gobbi C., Granziera C., Yaldizli Ö. (2018). Serum neurofilament as a predictor of disease worsening and brain and spinal cord atrophy in multiple sclerosis. Brain.

[28] Petzold A., Steenwijk M.D., Eikelenboom J.M., Wattjes M.P., Uitdehaag B.M. (2016). Elevated CSF neurofilament proteins predict brain atrophy: A 15-year follow-up study. Mult. Scler. J..

[29] Hyun J.-W., Kim Y., Kim G., Kim S.-H., Kim H.J. (2019). Longitudinal analysis of serum neurofilament light chain: A potential therapeutic monitoring biomarker for multiple sclerosis. Mult. Scler. J..

[30] Havrdova E., Arnold D.L., Cohen J.A., Hartung H.P., Fox E.J., Giovannoni G., Schippling S., Selmaj K.W., Traboulsee A., Compston D.A.S. (2017). Alemtuzumab CARE-MS I 5-year follow-up: Durable efficacy in the absence of continuous MS therapy. Neurology.

[31] Coles A.J., Cohen J.A., Fox E.J., Giovannoni G., Hartung H.P., Havrdova E., Schippling S., Selmaj K.W., Traboulsee A., Compston D.A.S. (2017). Alemtuzumab CARE-MS II 5-year follow-up: Efficacy and safety findings. Neurology.

[32] Green A.J., McQuaid S., Hauser S.L., Allen I.V., Lyness R. (2010). Ocular pathology in multiple sclerosis: Retinal atrophy and inflammation irrespective of disease duration. Brain.

[33] Petzold A., Balcer L.J., Calabresi P.A., Costello F., Frohman T.C., Frohman E.M., Martinez-Lapiscina E.H., Green A.J., Kardon R., Outteryck O. (2017). Retinal layer segmentation in multiple sclerosis: A systematic review and meta-analysis. Lancet Neurol..

[34] Costello F., Coupland S., Hodge W., Lorello G., Koroluk J., Pan Y.I., Freedman M.S., Zackon D.H., Kardon R.H. (2006). Quantifying axonal loss after optic neuritis with optical coherence tomography. Ann. Neurol..

[35] Talman L.S., Bisker E.R., Sackel D.J., Long D.A., Galetta K.M., Ratchford J.N., Lile D.J., Farrell S.K., Loguidice M.J., Remington G. (2010). Longitudinal study of vision and retinal nerve fiber layer thickness in multiple sclerosis. Ann. Neurol..

[36] Fisher J.B., Jacobs D.A., Markowitz C.E., Galetta S.L., Volpe N.J., Nano-Schiavi M.L., Baier M.L., Frohman E.M., Winslow H., Frohman T.C. (2006). Relation of Visual Function to Retinal Nerve Fiber Layer Thickness in Multiple Sclerosis. Ophthalmology.

[37] Garcia-Martin E., Rodriguez-Mena D., Herrero R., Almarcegui C., Dolz I., Martin J., Ara J.R., Larrosa J.M., Polo V., Fernández J. (2013). Neuro-ophthalmologic evaluation, quality of life, and functional disability in patients with MS. Neurology.

[38] Toledo J., Sepulcre J., Salinas-Alaman A., García-Layana A., Murie-Fernandez M., Bejarano B., Villoslada P. (2008). Retinal nerve fiber layer atrophy is associated with physical and cognitive disability in multiple sclerosis. Mult. Scler. J..

[39] Saidha S., Al-Louzi O., Ratchford J.N., Bhargava P., Oh J., Newsome S.D., Prince J.L., Pham D., Roy S., Van Zijl P. (2015). Optical coherence tomography reflects brain atrophy in multiple sclerosis: A four-year study. Ann. Neurol..

[40] Lambe J., Fitzgerald K.C., Murphy O.C., Filippatou A.G., Sotirchos E.S., Kalaitzidis G., Vasileiou E., Pellegrini N., Ogbuokiri E., Toliver B. (2021). Association of Spectral-Domain OCT With Long-term Disability Worsening in Multiple Sclerosis. Neurology.

[41] Pisa M., Guerrieri S., Di Maggio G., Medaglini S., Moiola L., Martinelli V., Comi G., Leocani L. (2017). No evidence of disease activity is associated with reduced rate of axonal retinal atrophy in MS. Neurology.

[42] Pisa M., Ratti F., Vabanesi M., Radaelli M., Guerrieri S., Moiola L., Martinelli V., Comi G., Leocani L. (2019). Subclinical neurodegeneration in multiple sclerosis and neuromyelitis optica spectrum disorder revealed by optical coherence tomography. Mult. Scler. J..

[43] Compston A., Coles A. (2008). Multiple sclerosis. Lancet.

[44] Sen M.K., Almuslehi M.S.M., Shortland P.J., Coorssen J.R., Mahns D.A. (2020). Revisiting the Pathoetiology of Multiple Sclerosis: Has the Tail Been Wagging the Mouse?. Front. Immunol..

[45] Hauser S.L., Cree B.A. (2020). Treatment of Multiple Sclerosis: A Review. Am. J. Med..

[46] Tintore M., Vidal-Jordana A., Sastre-Garriga J. (2019). Treatment of multiple sclerosis—Success from bench to bedside. Nat. Rev. Neurol..

[47] Amato M.P., Fonderico M., Portaccio E., Pastò L., Razzolini L., Prestipino E., Bellinvia A., Tudisco L., Fratangelo R., Comi G. (2020). Disease-modifying drugs can reduce disability progression in relapsing multiple sclerosis. Brain.

[48] Kalincik T., Diouf I., Sharmin S., Malpas C., Spelman T., Horakova D., Havrdova E.K., Trojano M., Izquierdo G., Lugaresi A. (2020). Effect of Disease-Modifying Therapy on Disability in Relapsing-Remitting Multiple Sclerosis over 15 Years. Neurology.

[49] Chalmer T.A., Baggesen L.M., Nørgaard M., Koch-Henriksen N., Magyari M., Sorensen P.S., The Danish Multiple Sclerosis Group (2018). Early versus later treatment start in multiple sclerosis: A register-based cohort study. Eur. J. Neurol..

[50] Buron M.D., Chalmer T.A., Sellebjerg F., Barzinji I., Christensen J.R., Christensen M.K., Hansen V., Illes Z., Jensen H.B., Kant M. (2020). Initial high-efficacy disease-modifying therapy in multiple sclerosis: A nationwide cohort study. Neurology.

[51] He A., Merkel B., Brown W., Ryerson L.Z., Kister I., Malpas C.B., Sharmin S., Horakova D., Havrdova E.K., Spelman T. (2020). Timing of high-efficacy therapy for multiple sclerosis: A retrospective observational cohort study. Lancet Neurol..

[52] Prosperini L., Mancinelli C.R., Solaro C.M., Nociti V., Haggiag S., Cordioli C., De Giglio L., De Rossi N., Galgani S., Rasia S. (2020). Induction Versus Escalation in Multiple Sclerosis: A 10-Year Real World Study. Neurotherapeutics.

[53] Di Filippo M., Anderson V.M., Altmann D.R., Swanton J.K., Plant G.T., Thompson A.J., Miller D.H. (2010). Brain atrophy and lesion load measures over 1 year relate to clinical status after 6 years in patients with clinically isolated syndromes. J. Neurol. Neurosurg. Psychiatry.

[54] Minneboo A., Jasperse B., Barkhof F., Uitdehaag B.M.J., Knol D.L., de Groot V., Polman C.H., Castelijns J.A. (2008). Predicting short-term disability progression in early multiple sclerosis: Added value of MRI parameters. J. Neurol. Neurosurg. Psychiatry.

[55] Branger P., Parienti J.-J., Sormani M.P., Defer G. (2016). The Effect of Disease-Modifying Drugs on Brain Atrophy in Relapsing-Remitting Multiple Sclerosis: A Meta-Analysis. PLoS ONE.

[56] Sormani M.P., Arnold D.L., de Stefano N. (2013). Treatment effect on brain atrophy correlates with treatment effect on disability in multiple sclerosis. Ann. Neurol..

[57] De Stefano N., Stromillo M.L., Giorgio A., Bartolozzi M.L., Battaglini M., Baldini M., Portaccio E., Amato M.P., Sormani M.P. (2016). Establishing pathological cut-offs of brain atrophy rates in multiple sclerosis. J. Neurol. Neurosurg. Psychiatry.

[58] Sprenger T., Kappos L., Radue E.-W., Gaetano L., Mueller-Lenke N., Wuerfel J., Poole E.M., Cavalier S. (2019). Association of brain volume loss and long-term disability outcomes in patients with multiple sclerosis treated with teriflunomide. Mult. Scler. J..

[59] De Stefano N., Tomic D., Radue E.W., Sprenger T., Meier D.P., Haring D., Sormani M.P. (2016). Effect of fingolimod on diffuse brain tissue damage in relapsing-remitting multiple sclerosis patients. Mult. Scler. Relat. Disord..

